# Genome-wide discovery of circulating cell-free DNA methylation biomarkers for colorectal cancer detection

**DOI:** 10.1186/s13148-023-01518-5

**Published:** 2023-07-27

**Authors:** Qingxiao Fang, Ziming Yuan, Hanqing Hu, Weiyuan Zhang, Guiyu Wang, Xishan Wang

**Affiliations:** 1 Colorectal Cancer Surgery Department, The Second Affiliated Hospital of Harbin Medical University, Harbin, Heilongjiang China; 2 Department of Colorectal Surgery, National Cancer Center/National Clinical Research Center for Cancer/Cancer Hospital, Chinese Academy of Medical Sciences and Peking Union Medical College, Beijing, China

**Keywords:** DNA methylation, Cell-free DNA, Colorectal cancer, Biomarker, Early detection

## Abstract

**Background:**

Colorectal polyp is known a precursor of colorectal cancer (CRC) that holds an increased risk for progression to CRC. Circulating cell-free DNA (cfDNA) methylation has shown favorable performance in the detection and monitoring the malignant progression in a variety of cancers.

**Results:**

To discover cfDNA methylation markers for the diagnosis of CRC, we first performed a genome-wide analysis between eight CRC and eight polyp tissues using the Infinium HumanMethylationEPIC BeadChip. We identified 7008 DMCs, and after filtering, we validated 39 DMCs by MethylTarget sequencing in 62 CRC and 56 polyp tissues. A panel of four CpGs (cg04486886, cg06712559, cg13539460, and cg27541454) was selected as the methylation marker in tissue by LASSO and random forest models. A diagnosis prediction model was built based on the four CpGs, and the methylation diagnosis score (md-score) can effectively discriminate tissues with CRC from polyp patients (AUROC > 0.9). Finally, the cg27541454 was confirmed hypermethylated in CRC (AUC = 0.85) in the plasma validation cohort.

**Conclusions:**

Our findings suggest that the md-score could robustly detect CRC from polyp tissues, and cg27541454 may be a promising candidate noninvasive biomarker for CRC early diagnosis.

**Supplementary Information:**

The online version contains supplementary material available at 10.1186/s13148-023-01518-5.

## Introduction

Colorectal cancer is the third most common malignant tumor in the world, with a high degree of malignancy. About 600,000 people die of colorectal cancer every year, and most patients are already at an advanced stage of the disease when symptoms appear [1]. Abnormal expression or structure of intracellular core regulatory pathway molecules lead to cell growth and metabolism disorders, which promote normal intestinal mucosa to transform into intestinal adenoma or intestinal polyps, and then develop into malignant tumors with the gradual accumulation of abnormal molecules [2, 3]. For adenomas larger than 1 cm, the cumulative risk of diagnosing cancer at the polyp site at 5, 10, and 20 years was 2.5%, 8%, and 24%, respectively [4]. Therefore, there is an urgent need for biomarkers that can detect early CRC in the context of polyp.

Although the detection rate of traditional examination methods is increasing with the progress of imaging level and the popularization of colonoscopy, the diagnosis of early and asymptomatic colon cancer is still not satisfactory. Carcinoembryonic antigen (CEA) is the most characteristic serological marker of colorectal cancer, while the sensitivity of serum CEA is generally low [5, 6]. In recent years, with the deepening of epigenetics research, the role of DNA methylation in the occurrence and development of colorectal cancer has gradually attracted more attention. Studies have shown that the frequency of abnormal DNA methylation in colorectal cancer is higher and earlier than genetic changes [7]. Circulating cell-free DNA (cfDNA) is an extracellular nucleic acid fragment released into the plasma by cell necrosis, apoptosis or activity [8]. The amount of cfDNA has been reported to be higher in tumors than in healthy individuals, and it is shown to be related to tumor size and clinical stage [9–11]. In recent years, plasma cell-free DNA methylation has shown favorable performance in the early detection of a variety of cancers [12–14]. Circulating cell-free DNA methylation has rapidly emerged as an effective noninvasive blood biomarker for early cancer detection, monitoring tumor progression and treatment response [15].

In this study, we performed a genome-wide 5mC profiling by EPIC BeadChip in colorectal tissues, comprised of samples from tumor and polyp, to identify specific CpGs that differentiate between these two disease states. After experimental validation of differential methylation CpGs in a larger cohort, four methylation markers were selected, and a diagnostic model was developed and tested in CRC tissues. Although the model did not have nearly performance as well in cfDNA, we showed cg27541454 that may serve as a cfDNA methylation biomarker for CRC early detection.

## Results

### Clinical characteristics of samples

A total of 65 CRC and 56 polyp patients were enrolled in this study to discovery and validate CRC methylation markers. The EPIC tissue discovery cohort consisting of eight CRC and eight polyp was used to perform genome-wide methylation profiling. The MethylTarget sequencing tissue validation cohort included 62 CRC and 56 polyp samples. The age was relatively balanced between CRC and polyp patients (median, 60.5 years vs. 60.5 years). The 62 primary tissues from CRC included 32 patients with stage I/II and 30 patients with stage III/IV. The MethylTarget sequencing plasma validation samples were collected from 20 CRC and 20 polyp patients. The CRC patients comprised of eight stage I/II and 12 stage III/IV. The median age was 59.5 years in CRC patients and 60.5 years in polyp patients. Detailed patient characteristics are summarized in Table 1.

**Table 1:** Clinical characteristics of tissue and plasma cohorts

Sample	Tissue		Plasma	
Characteristics	polyp	CRC	polyp	CRC
Total (n)	56	62	20	20
*Gender*				
Male	40	45	14	17
Female	16	17	6	3
*Age (years)*				
Median	60.7 (37–94)	60.5 (32–81)	60.5 (50–73)	59.5 (32–74)
≥ 50	51	53	20	16
< 50	5	9	0	4
*Stage*				
I + II	NA	32	NA	8
III + IV	NA	30	NA	12
*Lymph node metastasis*				
No	NA	35	NA	10
Yes	NA	27	NA	10
*Microsatellite instability*				
MSS	NA	58	NA	17
MSI	NA	0	NA	0
NA	NA	4	NA	3
*Tumor site*				
Right-sided	11	9	5	4
Left-sided	22	13	10	4
Rectum	23	40	5	12
*Differentiation grade*				
Highly	NA	1	NA	0
Moderately	NA	56	NA	18
Poorly	NA	5	NA	2
*Vascular invasion*				
No	NA	46	NA	14
Yes	NA	13	NA	3
NA	NA	3	NA	3
*Tumor size*				
≥ 5CM	NA	27	NA	6
< 5CM	NA	35	NA	14
*Polyp size*				
≥ 1CM	20	NA	4	NA
< 1CM	36	NA	16	NA

### Genome-wide discovery of differential methylation from primary tissues

**Fig. 1:**
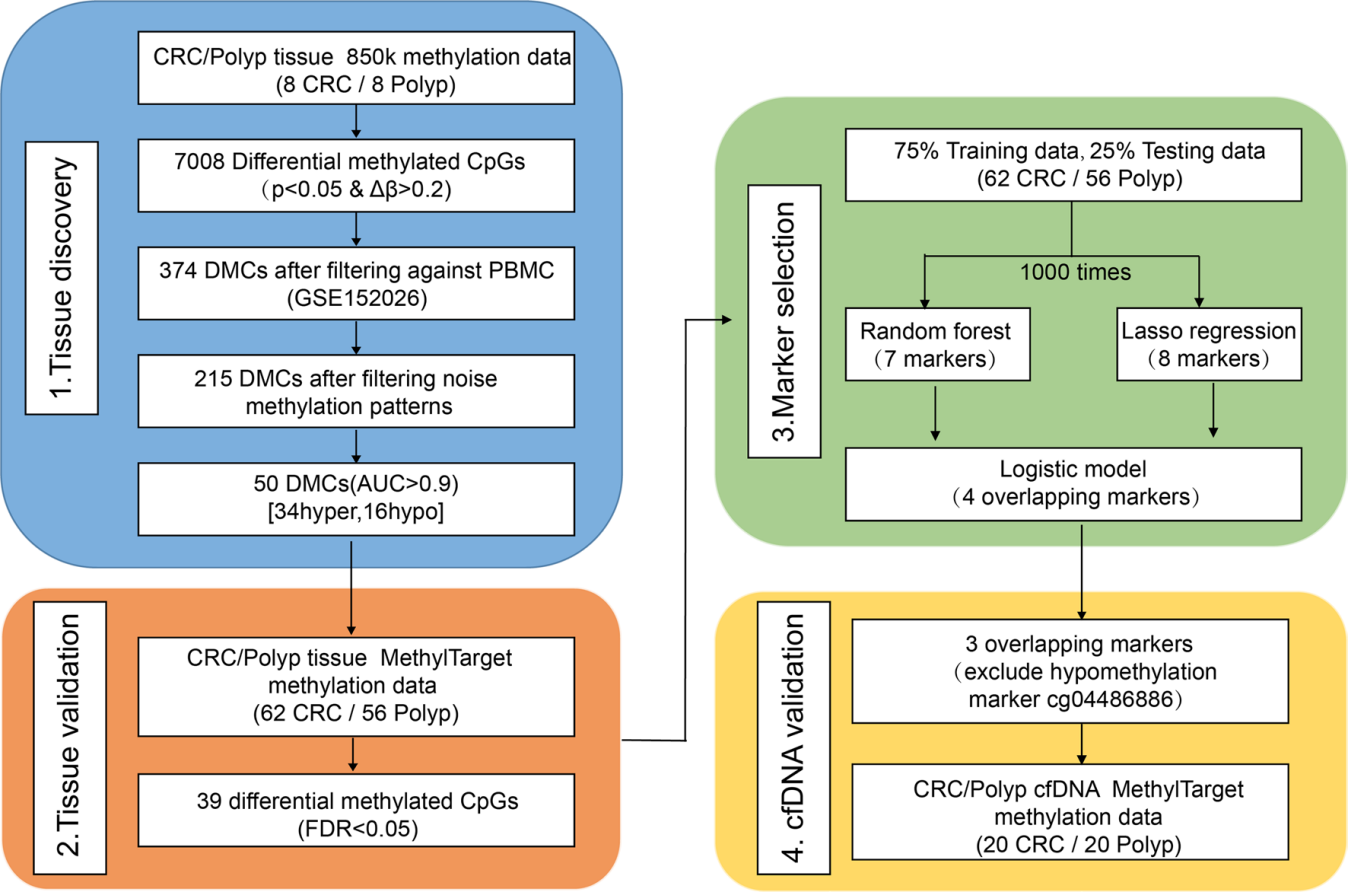
Workflow of the study. A multistep analysis to identify cfDNA methylation-based biomarkers for CRC detection in polyp patients. First, starting with genome-wide methylation analysis in primary tissues to discover differentially methylated CpGs and then validated their performance in a larger tissue cohort. Random forest and LASSO models were applied to the training set of tissue validation cohort in order to refine methylation markers. The methylation levels of three hypermethylated markers were measured in the cfDNA validation cohort

This study aimed to identify methylation cfDNA detection markers for CRC through the following sections: tissue discovery, tissue validation, marker selection and cfDNA validation (Fig. 1).

**Fig. 2:**
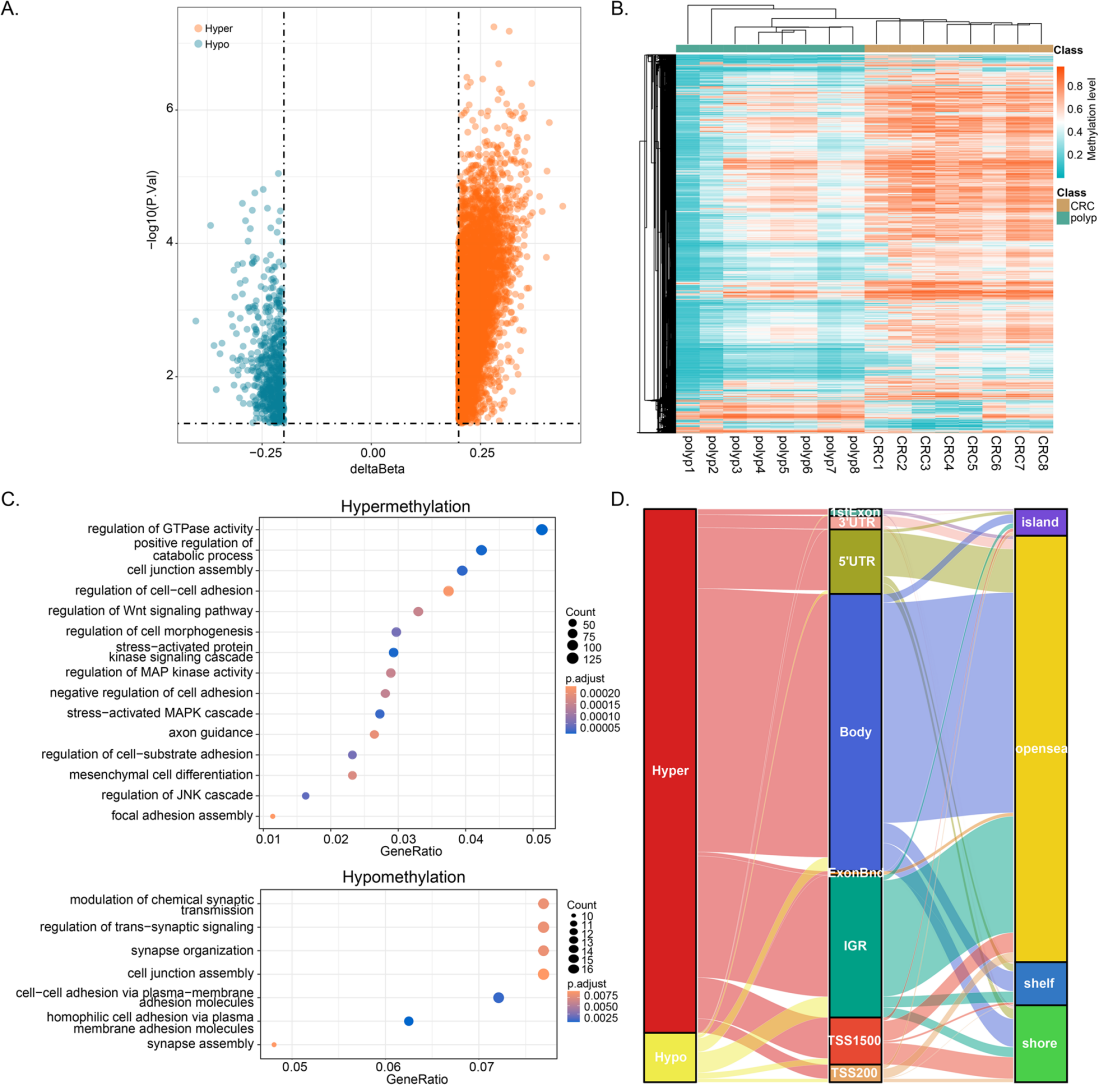
Identification of differentially methylated CpGs between CRC and polyp. **A** Volcano plots illustrating the hyper- and hypo-DMCs. **B** Heatmap illustrating the DMCs between CRC (*n* = 8) and polyp (*n* = 8). **C** Gene ontology enrichment analysis of genes with hyper- and hypo-DMCs. **D** Sankey plot of the hyper- and hypo-DMCs

We first performed HumanMethylationEPIC array-based DNA methylation analysis on eight CRC tissues and eight polyp tissues (Additional file 1: Figure S1). A total of 7008 differential methylated CpGs (DMCs) were identified between CRC and polyp (Fig. 2A). The unsupervised hierarchical clustering also showed different methylation patterns in these DMCs between CRC and polyp patients (Fig. 2B). Of these DMCs, 6404 (91.38%) DMCs showed higher methylation levels in CRC tissues (defined as hyper-DMCs) and 604 (8.62%) DMCs showed lower methylation levels in CRC tissues (defined as hypo- DMCs). GO enrichment analysis showed that the genes with hyper-DMCs were enriched in the biological processes involved in regulation of GTPase activity, cell–cell adhesion and Wnt signaling pathway (Fig. 2C). The genes with hypo-DMCs enriched in homophilic cell adhesion via plasma membrane adhesion molecules and regulation of trans-synaptic signaling. As shown in the Sankey plot (Fig. 2D), most of the hyper- DMCs were located in intergenic region (IGR), body and 5’UTR, and hypo-DMCs were also exhibited a higher proportion in intergenic region (IGR) (42.05%). The hyper-DMCs were related with opensea (75.61%).

### MethylTarget sequencing validation for differential DNA methylation in tissue

**Fig. 3:**
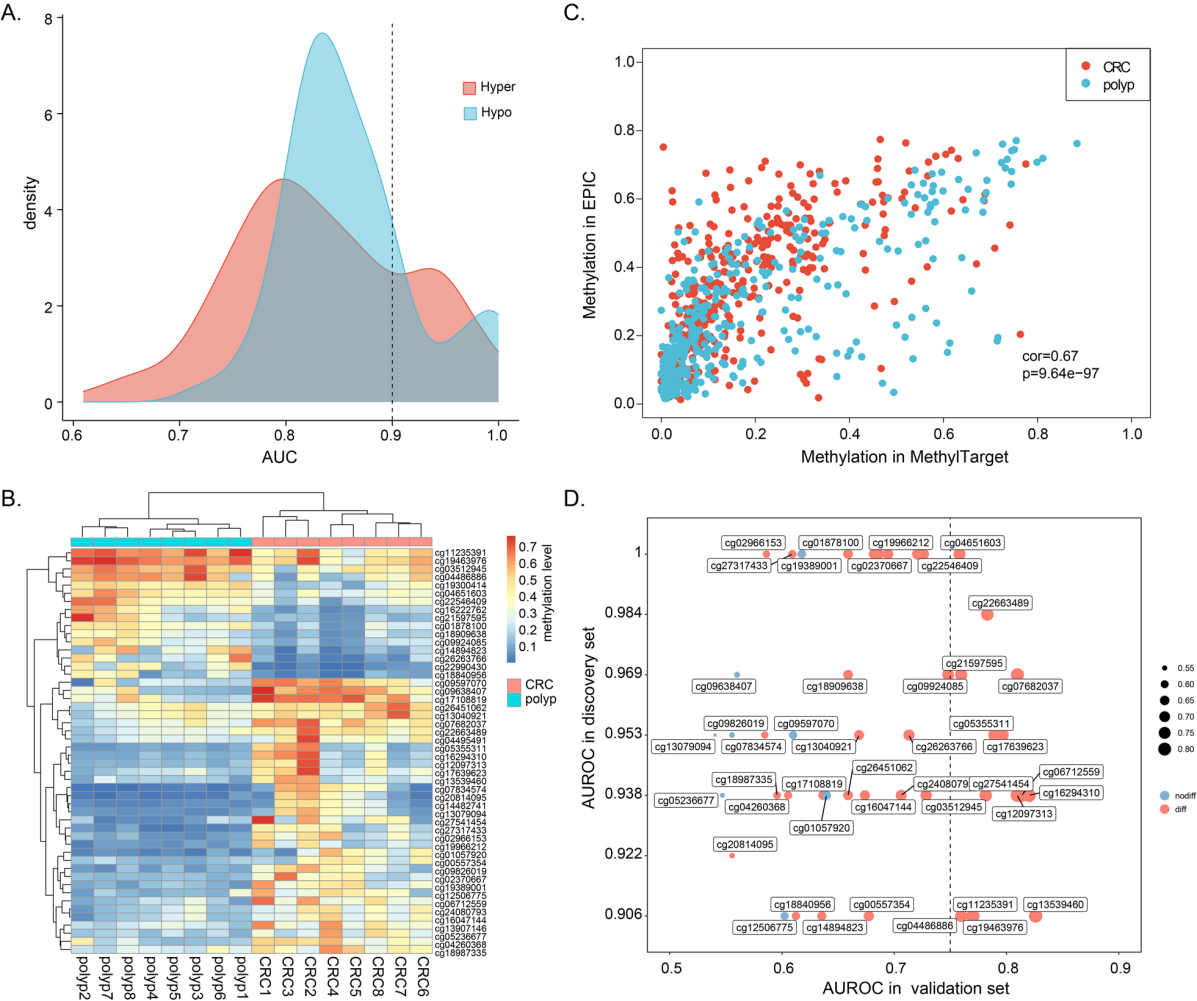
Validation DNA methylation markers in the tissue validation cohort. **A** Area under the receiver operating characteristic curves for CpGs identified in the tissue discovery cohort. **B** Heatmap of 50 differentially methylated CpGs between CRC and polyp tissue. **C** Scatterplot of methylation level consistency examined by MethylTarget sequencing and EPIC array. **D** Scatterplot of AUROCs from the 47 CpGs in the tissue discovery set (y-axis) and in the tissue validation set (x-axis)

Since cfDNA can be derived from normal leukocytes and cancer cells, we excluded the positive CpGs (*β* > 0.2) in blood tests to reduce the possibility of false positive in detection of cancer-derived methylation signal, and we reserved 374 DMCs of 7008 DMCs. Through filtering the noise methylation patterns, 215 DMCs were used for further analysis. Then, area under the receiver operating characteristic curve (AUROC) analysis was performed for each DMC to evaluate its performance in distinguishing between CRC and polyp. The results showed that 50 of the 215 (23.26%) CpGs had a strong discriminative power in the discovery tissue cohort, including 34 hyper-DMCs and 16 hypo-DMCs (AUROC > 0.9, Fig. 3A, Additional file 1: Table S1). We performed PCA on all samples and revealed that the tumor patients localized to a distinct cluster independent from polyp patients (Additional file 1: Figure S2). Moreover, the unsupervised hierarchical clustering of 50 CpGs confirms that the two disease states were significantly different (Fig. 3B).

Next, we performed MethylTarget sequencing on the 50 CpGs and obtained the methylation profiling of 47 CpGs (3 CpGs failed in the primer optimization) in the tissue validation cohort (Additional file 2: Table S2), which composed of 62 CRC and 56 polyp samples. We observed a high consistency between EPIC and MethylTarget in both CRC and polyp samples (Fig. 3C). The differential methylation analysis confirmed 39 CpGs were also differentially methylated between two disease states (Wilcoxon rank-sum test, FDR < 0.05). Nevertheless, most CpGs were unable to distinguish well between CRC and polyp alone in the tissue validation cohort (using a threshold of 0.75, Fig. 3D).

### Identification of DNA methylation markers of CRC diagnosis in tissue

**Fig. 4:**
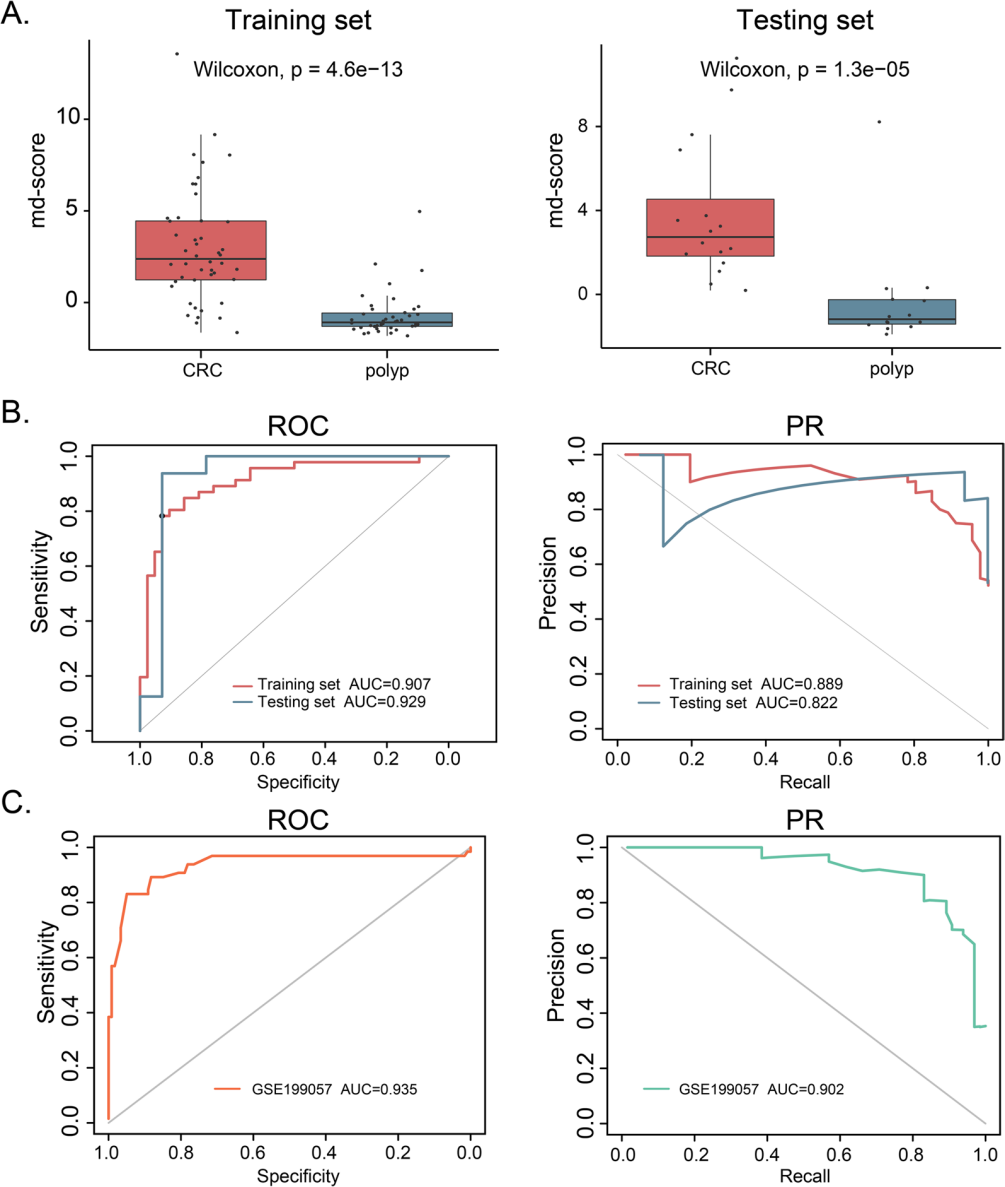
Diagnostic power of methylation markers and methylation diagnosis score (md-score) in tissue. **A** Boxplots of md-score for CRC and polyp in the training set and testing set. **B** ROC curves and PR curves of md-scores in the training set and testing set. **C** ROC curve and PR curve of md-score in the independent validation set (GSE199057)

To further refine CpGs that can distinguish CRC from polyp, we applied two feature selection methods in the 39 differentially methylated CpGs validated in the MethylTarget sequencing. To assess the stability of features and selected important features, we performed the analysis of least absolute shrinkage and selection operator (LASSO) and random forest (RF) for 1000 times. We obtained four overlapping markers (cg04486886, cg06712559, cg13539460, and cg27541454) from eight markers in the LASSO model and seven markers in the RF model (Table 2, Additional file 1: Figure S3A). We showed the diagnostic performance of these four markers with ROC curves, and the corresponding AUCs were 0.804, 0.829, 0.833 and 0.827, respectively (Additional file 1: Figure S3B). Then, we constructed a logistic regression model using these four markers and calculated a methylation diagnosis score (md-score) for each sample according to the markers’ regression coefficients multiplied by their methylation values. The md-scores were significantly differential between CRC and polyp patients both in the training set and the testing set (Fig. 4A). In addition, compared with each individual marker, the md-score demonstrated higher sensitivity and specificity for CRC diagnosis (AUROC = 0.907 vs. AUPRC = 0.889 for the training set, AUROC = 0.929 vs. AUPRC = 0.822 for the testing set).

**Table 2:** List of the genomic locations of four methylation markers and their corresponding genes

CpG	Position (hg19)	Gene	Region	DMC
cg04486886	chr5:56,784,195	ACTBL2	Intergenic	Hypo-DMC
cg06712559	chr1:968,395	AGRN	Body	Hyper-DMC
cg13539460	chr19:46,854,076	PPP5C	Body	Hyper-DMC
cg27541454	chr1:975,551	AGRN	Body	Hyper-DMC

**Fig. 5:**
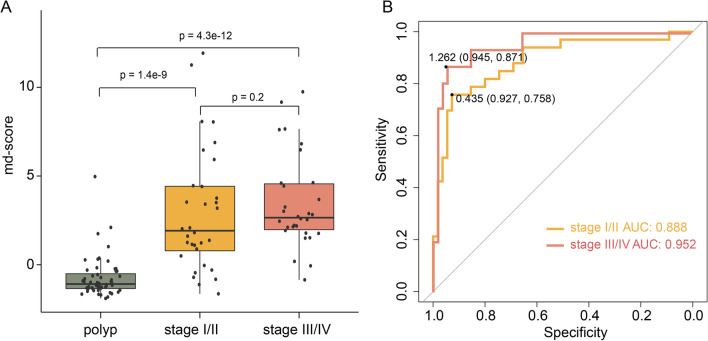
Diagnostic power of methylation diagnosis score (md-score) in CRC with different clinical stages. **A** The md-scores of polyp and different stages of CRC. **B** ROC curves of md-score for distinguishing CRC with stage I/II and stage (III/IV) from polyp, respectively

To evaluate the universality of md-score, we analyzed the methylation data of an independent validation cohort (GSE199057) measured by EPIC array, including 76 tumor samples from CRC patients, 78 normal samples from CRC patients, and 68 normal samples from non-CRC patients. The results revealed that the md-score can effectively distinguish CRC from normal samples (Fig. 4C and Additional file 1: Figure S4). Although the md-score value increased with the increasing malignancy of tumors, there was no significant difference in md-score values between stage I/II and stage III/IV CRC patients (*p* = 0.2, Fig. 5A), indicating that md-score value was not affected by the patient stage. ROC curve analysis based on md-score value to distinguish early/late CRC and polyp patients revealed that it had high predictive ability and robustness in patients with different stages. Especially for stage III/IV patients, the AUROC value was 0.952. Using the best cutoff values, the sensitivity and specificity were 0.758 and 0.927 (AUROC = 0.888), respectively, for discriminating stage I/II CRC from polyp. And the sensitivity and specificity were 0.871 and 0.945 (AUROC = 0.952), respectively, for discriminating stage III/IV CRC from polyp (Fig. 5B).

### Circulating free DNA-based validation for CRC and polyp

**Fig. 6:**
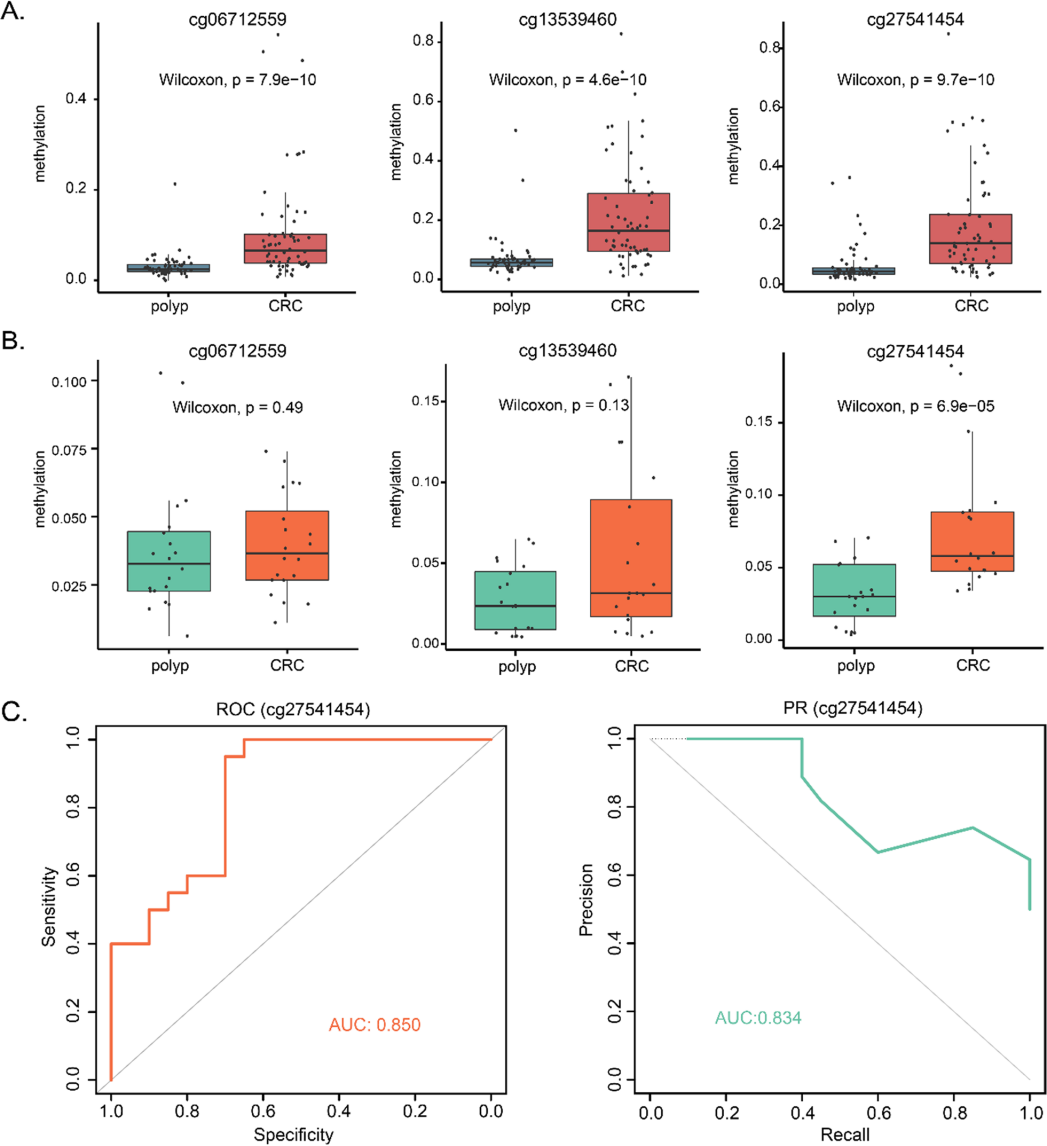
Methylation level of methylation markers in plasma. **A** Three of four methylation markers were hypermethylated in CRC tissue. **B** Boxplots of cfDNA methylation levels of three methylation markers in CRC and polyp plasma. **C** The ROC curve and PR curve of cg27541454 cfDNA methylation

With the ultrasensitive MethylTarget sequencing, we can measure the methylation status of lower-input cfDNA while maintaining sufficient diversity and sensitivity. The quality control of cfDNA was evaluated by Bioanalyzer 2100 (Additional file 1: Figure S5). Since the hypermethylation events might be preferred as biomarkers due to the desire for a gain of ‘signal’ [13], we only examined the methylation levels of three markers with hypermethylation in CRC identified in the tissue validation cohort (Fig. 6A and Additional file 1: Figure S6). The cfDNA validation cohort consisted of 20 CRC and 20 polyp samples. Remarkably, cg27541454 was differentially methylated between CRC plasma and polyp plasma (*p* = 6.9e-05, Fig. 6B). The AUROC and AUPRC of cg27541454 was 0.850 and 0.834, revealed that tissue-derived CpGs can also perform robustly in plasma cfDNA (Fig. 6C). In addition, the methylation level of cg27541454 can distinguish both stage I/II and stage III/IV CRC patients from polyps (Additional file 1: Figure S7). Together, cg27541454 may be served as a promising candidate noninvasive biomarker for CRC early diagnosis.

## Discussion

Colorectal cancer (CRC) is one of the most prevalent cancers and leading causes of cancer-related deaths in the world. Most colorectal tumors arise from adenomas that beginning as polyps, and the pathological progression of CRC is closely related to polyp [7, 16, 17]. Although CRC could be relatively easily detected by colonoscopy, the development of novel biomarkers for CRC detection are needed due to its disadvantages such as expensive, invasive and bleeding risk [18, 19]. Compared with normal mucosal, it is especially urgent to find blood-based biomarkers with sensitive, specific and noninvasive for early diagnosis of CRC in polyp patients, which is vital to improve the diagnosis and prognosis of CRC. Since methylated cfDNA is more stable in blood compared to mutated ctDNA [20], it can serve as a potential biomarker for CRC diagnosis that distinguish between CRC and polyp patients.

In this study, we first performed a genome-wide differential methylation analysis from primary colorectal cancer and polyp tissues based on EPIC BeadChip data. By filtering the probes, we screened 50 differentially methylated CpGs with highly diagnostic efficiency between CRC and polyp. Through target sequencing of bisulfate-converted DNA, we validated these DMCs in a larger cohort and selected four markers to build a methylation diagnosis score (md-score) that had a high accuracy for discrimination between CRC and polyp or healthy controls. Three of these markers were hypermethylated in CRC than polyp, which were further used to validate in plasma cfDNA. However, we only observed one methylation marker cg27541454 also showed significant hypermethylation in CRC than polyp in plasma cfDNA. Plasma cfDNA is derived from multiple tissues and cell types, including normal cells, tumor cells, and apoptosis and necrosis of other tissue cells. The mixing of these different sources of cfDNA may mask signals of differential methylation sites for tumor tissue. The methylation changes at cg27541454 may be more pronounced or detectable in plasma, while the other two CpG sites may have small changes in plasma methylation levels that are insufficient to show a difference. Therefore, our results indicated that cg27541454 can be used as a candidate noninvasive marker for CRC early detection screening.

Several limitations of this study should not be ignored. First, our study was limited by a relatively small cohort to discovery and validate methylation markers in tissue DNA and plasma cfDNA. While our identified CpGs can robustly segregate CRC from polyp patients, cfDNA-based data from a larger cohort is needed in subsequent analysis to further verify the clinical utility as a noninvasive colorectal cancer marker. Second, as the limitations of cfDNA methylation detection technology and the amount of blood obtained from patients, we identified circulating cell-free DNA methylation biomarkers from primary colorectal tissue instead of discovering markers from genome-wide methylation data derived directly from plasma cfDNA. Although our strategy of indirectly screening cfDNA methylation markers from tissues was feasible, we failed to find more high performing blood-based noninvasive biomarkers to distinguish between cancer and polyp states.

In summary, we established a diagnostic model containing four markers (cg04486886, cg06712559, cg13539460, and cg27541454) based on methylation patterns in tissue, which serves as a reliable approach for the early diagnosis of CRC. The cg27541454 holds great clinical potential in early noninvasive diagnosis and screening of CRC.

## Materials and methods

### Patient enrollment and sample acquisition

Blood and tissue samples of patients with colorectal cancer and colorectal polyps were collected during August 2020 through January 2021. A total of 62 CRC patients and 56 colorectal polyp patients had been confirmed by colonoscopy and histology. Sixty-two specimens of colorectal carcinoma tissues were obtained by surgical resection, and 56 specimens of adenomatoid polyp were taken during endoscopic examination. Dissect tissue sample quickly and freeze in liquid nitrogen. Whole blood (5 ml) was obtained from 20 patients with colorectal carcinoma and the 20 adenomatoid polyp patients drawn 1–3 days prior to surgery and stored in anticoagulant blood collection vessels for transported under refrigerated conditions. The samples were centrifuged in the tube at 2000 g at 4 °C for 10 min to separate the plasma and cell components. The supernatant was transferred to a new centrifuge tube, and then centrifuged at 12,000 g at 4 °C for 10 min. The supernatant obtained was plasma. Plasma samples were collected and stored at − 80 °C. All plasma and tissue samples were taken from the General Surgery Department of the First Affiliated Hospital of Harbin Medical University and the Digestive Department of Harbin Second Hospital.

To verify the accuracy and reliability of our methylation markers and the diagnosis model, we adopted a CRC cohort for validation. The human methylation EPIC array of samples was available from Gene Expression Omnibus (http://www.ncbi.nlm.nih.gov/geo/; accession number: GSE199057).

### DNA extraction of tissue and plasma

Tissue DNA was isolated using the QIAamp DNA Blood and Tissue Kit as per manufacturer instructions (QIAamp DNA Blood and Tissue Kit, Qiagen®, Germantown, MD). Nanodrop 2000 is used to detect the quality of genomic DNA and Invitrogen Qubit 3.0 Spectrophotometer is used to quantify the purified DNA.

Circulating cell-free DNA (cfDNA) was extracted from the plasma using MagMAX™ CellFree DNA Isolation Kit (Thermo Fisher, Cat# A29319) according to the manufacturer's instructions. For each patient we used 2 ml of plasma for cfDNA extraction and recovered cfDNA in 20 μl of elution buffer. Repeated freezing and thawing of plasma were avoided to prevent cfDNA degradation and gDNA contamination from white blood cells (WBCs). The concentration and quality of cfDNA were assessed by Bioanalyzer 2100 (Agilent Technologies), and cfDNA samples with high molecular weight DNA would be excluded from the study. cfDNA was stored at − 20 °C until further use.

### MethylTarget library preparation and sequencing

MethylTarget™, an NGS-based multiple targeted CpG methylation detection method developed by Genesky BioTech (Shanghai, China), was carried out as previously described [21, 22]. Tissue DNA and blood cfDNA were subjected to bisulfite conversion using EZ DNA Methylation-Gold™ kit (ZYMO RESEARCH) according to the manufacturer’s instructions. Using the optimized multiplex PCR primer panel, the transformed genomic DNA was used as the template for multiplex PCR amplification. PCR products were separated by agarose electrophoresis and purified using TIANgel Midi Purification Kit (TIANGEN). Libraries from different samples were quantified, pooled, and sequenced on the Illumina sequencer according to the manufacturer’s protocols, with 2 × 150 bp paired-end mode.

### Methylation data processing and differential analysis

The genome-wide DNA methylation was quantified using the Illumina Infinium HumanMethylationEPIC BeadChip (850 k). The tissue discovery cohort included eight CRC and eight polyp samples. The “ChAMP” package was used to extract the probe signal strength from the original.dat files of the methylation chip and perform differential methylation analysis. The CpGs with methylation Δ*β* > 0.2 and *p* value < 0.05 were identified as differentially methylated CpGs (DMCs). To increase the sensitivity and specificity of cfDNA methylation in the subsequent analysis, we removed hypermethylation CpGs in normal blood from DMCs. The normal leukocyte 850 k methylation data were obtained from GEO (GSE152026), including 934 DNA samples isolated from blood for schizophrenia cases and controls [23]. We considered that the CpGs of mean methylation level more than 0.2 in normal leucocytes were positive CpGs, and we excluded them from DMCs to minimize the risk of false positivity in blood tests.

In the MethylTarget sequencing, we designed probes corresponding to the DMCs and selected probes with at least 3 CpGs located within its 25 bp upstream and downstream. After filtering noise methylation patterns, the MethylTarget sequencing of 47 CpGs was performed on tissue DNA from 62 CRC and 56 polyp samples, which served as tissue validation set. In addition, we performed MethylTarget sequencing on plasma cfDNA from 20 CRC and 20 polyp samples, which served as cfDNA validation set. Wilcoxon rank-sum test was applied to differential methylation analysis of MethylTarget methylation data.

### Tissue methylation markers selection

Samples in the tissue validation cohort were randomly split into training set and testing set with a 3:1 ratio. We applied two feature selection methods in the training set to identify methylation markers for discriminating CRC and polyp patients. The random forest model was run through *R* package “randomForest” with “ntree” set to 500. The importance score of each feature was computed by the Gini index value as the mean decrease in accuracy and selected top 10 important markers. Least absolute shrinkage and selection operator (LASSO) model was run through *R* package “glmnet.” The tuning parameter (*λ*) selection in the LASSO model was used by fivefold cross-validation via minimum criteria and then selected the features with coefficients > 0. We repeated above processes 1000 times and selected the markers as the important features more than 700 times in the random forest model and the markers with occurrence frequency more than 700 times in the LASSO model. The overlapping markers selected by the two methods were considered as tissue methylation markers.

### Construction of a diagnosis model

A logistic regression model was constructed by using four overlapping methylation markers as the covariates with the training dataset. We then built a methylation diagnosis score (md-score) according the coefficients multiplying the markers’ methylation values. The predictability of the md-score was evaluated by area under receiver operating characteristic curve (AUROC) and precision–recall curve (AUPRC) in the training set, testing set and external independent validation set.

## Supplementary Information

**Additional file 1. ** Supplementary figures and Table S1.

**Additional file 2.** Supplementary Table S2 The sequence information on 47 CpGs in the range of 25bp upstream and downstream.

**Additional file 3.**  Supplementary Table S3 The processed tissue MethylTarget sequencing data.

**Additional file 4.** Supplementary Table S4 The processed cfDNA MethylTarget sequencing data.

## Data Availability

Human Methylation EPIC array data generated in our study have been uploaded to GEO under the accession number GSE220160. The processed MethylTarget sequencing data was provided in the Additional file 3: Table S3 and Additional file 4: Table S4.
