# Supplementary material for: Genome-wide discovery of circulating cell-free DNA methylation biomarkers for colorectal cancer detection
Source: Clin Epigenetics. 2023 Jul 27;15:119. doi: 10.1186/s13148-023-01518-5 (PMC10375686; doi:10.1186/s13148-023-01518-5)
Supplement: Supplementary file 1 — Additional file 1. Supplementary figures and Table S1. [file 13148_2023_1518_MOESM1_ESM.docx]

**Genome-wide discovery of circulating cell free DNA methylation biomarkers for colorectal cancer detection**

Qingxiao Fang^1^, Ziming Yuan^1^, Hanqing Hu^1^, Weiyuan Zhang^1^, Guiyu Wang^1🖂^, Xishan Wang^1,2🖂^

1. Colorectal Cancer Surgery Department, The Second Affiliated Hospital of Harbin Medical University, Harbin, Heilongjiang, China

1. Department of Colorectal Surgery, National Cancer Center/National Clinical Research Center for Cancer/Cancer Hospital, Chinese Academy of Medical Sciences and Peking Union Medical College, Beijing, China

🖂Corresponding authors: Xishan Wang (email: wxshan1208@126.com); Guiyu Wang (email: guiywang@hrbmu.edu.cn)

**Supplementary figures**


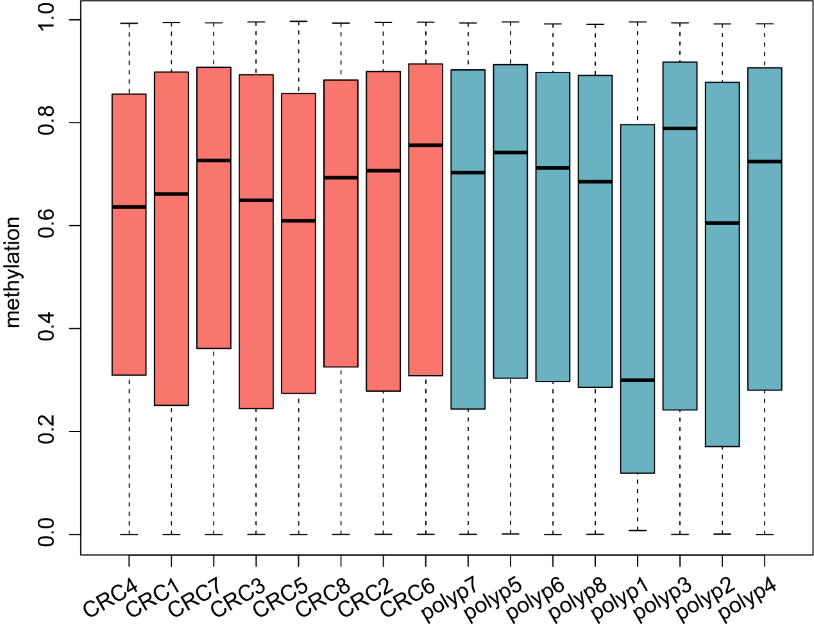


**Figure S1. Overall methylation level bar charts (as beta values) for individual CRC and polyp sample.**


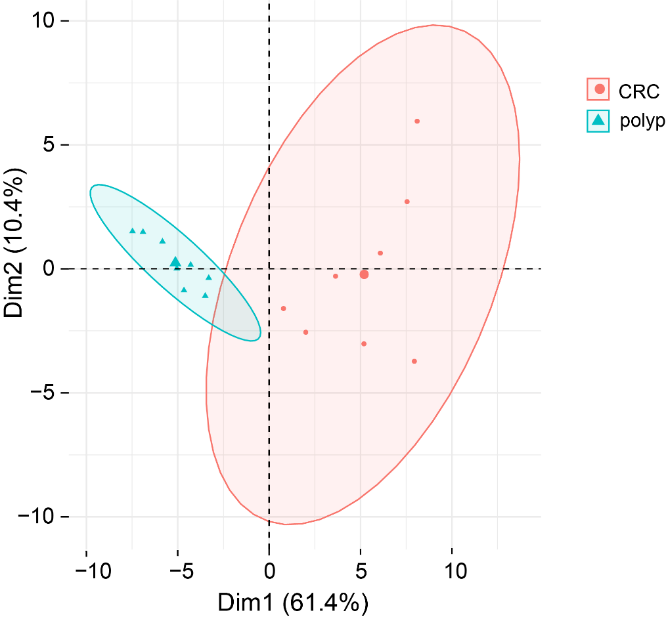


**Figure S2. PCA plot of 50 DMCs on 8 CRC and 8 polyp tissue samples.**


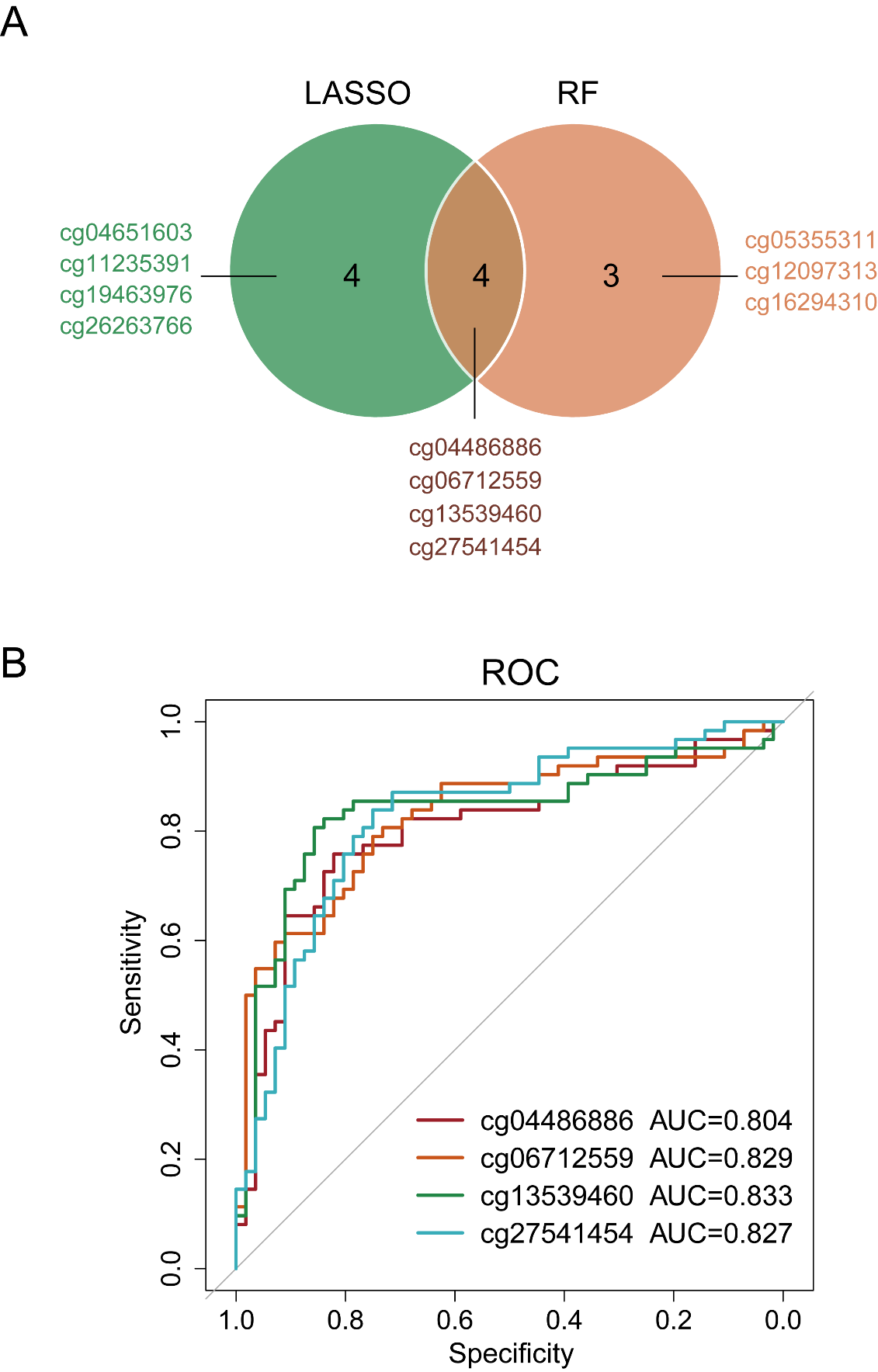


**Figure S3. Four methylation markers selected from tissue validation dataset.** (A) Overlapping markers of LASSO method and random forest method. (B) ROC curves of md-score in the tissue validation set (GSE199057).


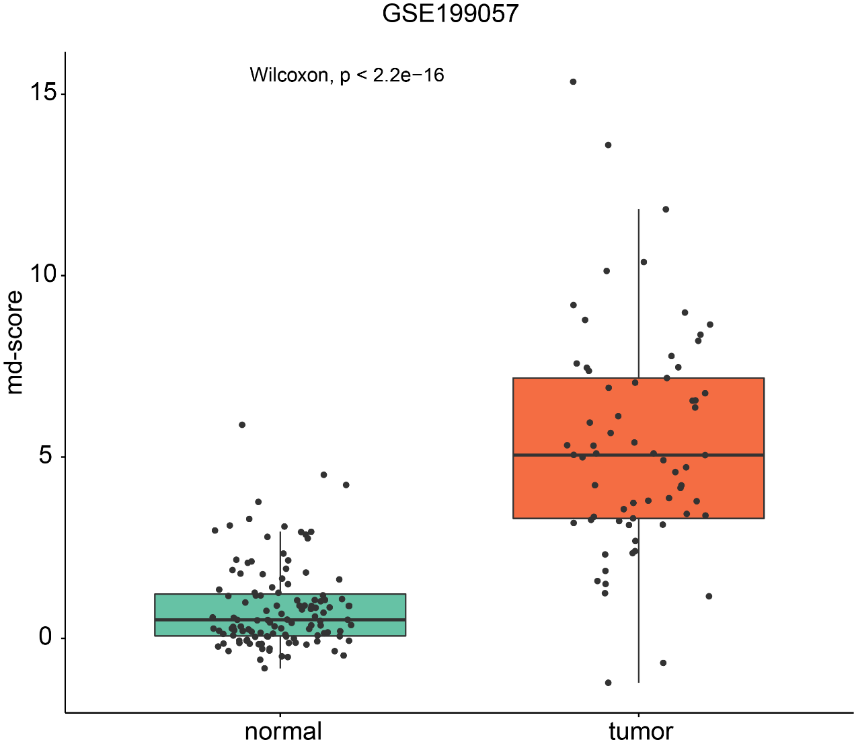


**Figure S4. Boxplots of md-score for CRC and normal in the independent validation set (GSE199057).**

**
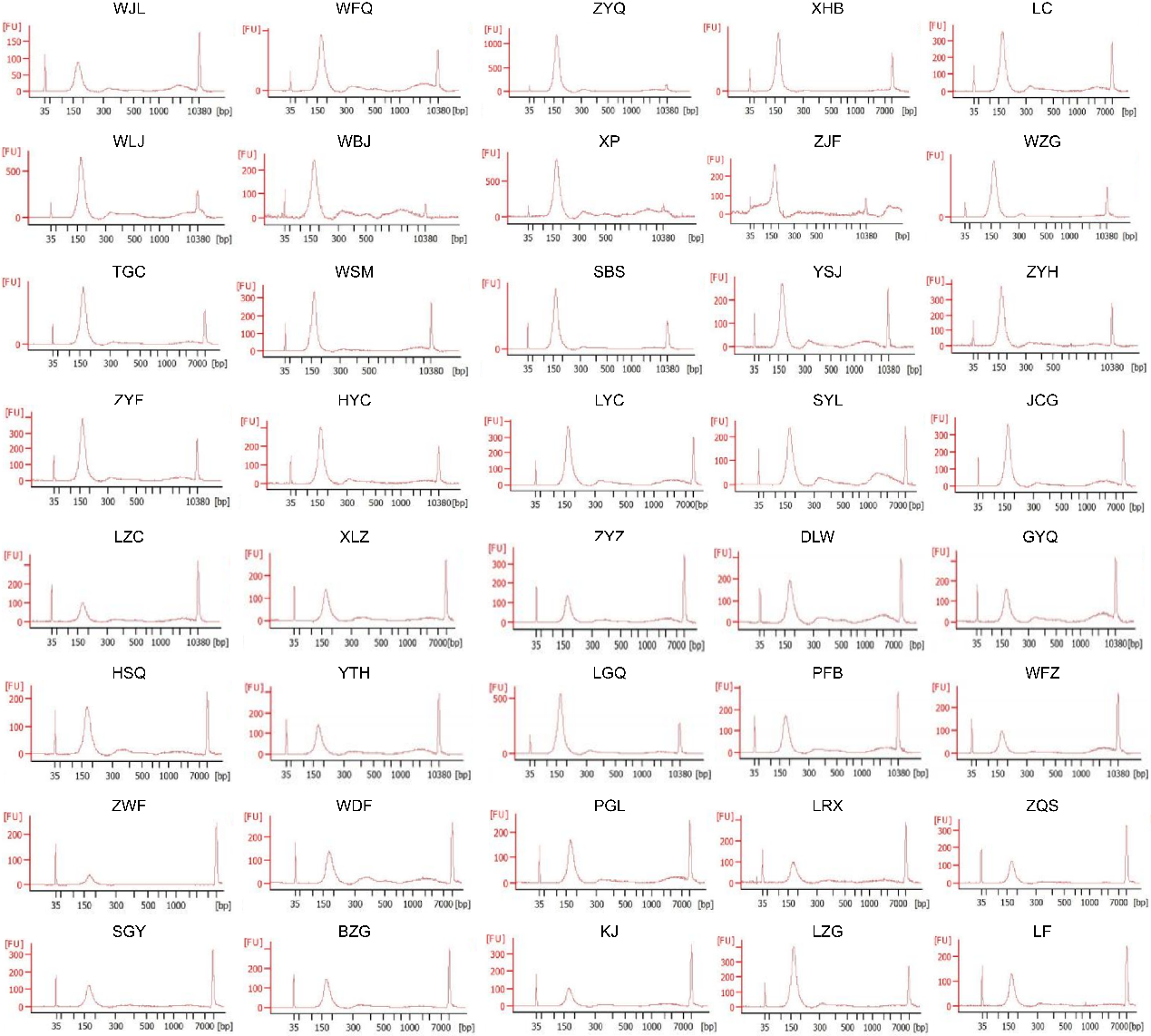
**

**Figure S5. The quality of cfDNA of 20 CRC and 20 polyp patients assessed by Bioanalyzer 2100.**


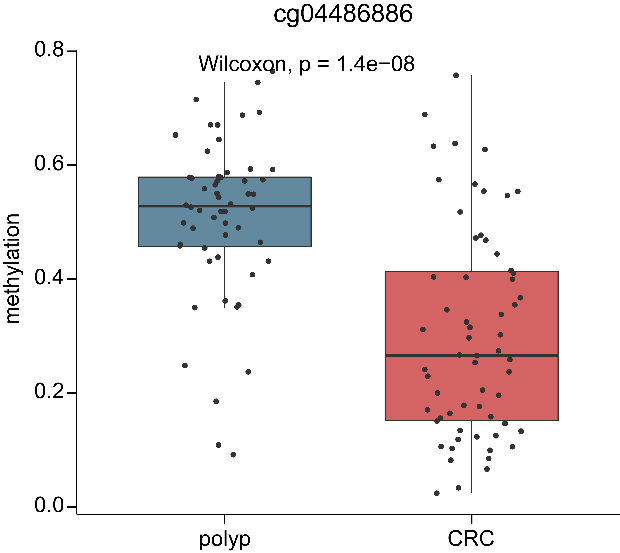


**Figure S6. The methylation levels of cg04486886 in polyp and CRC in tissue validation set.**


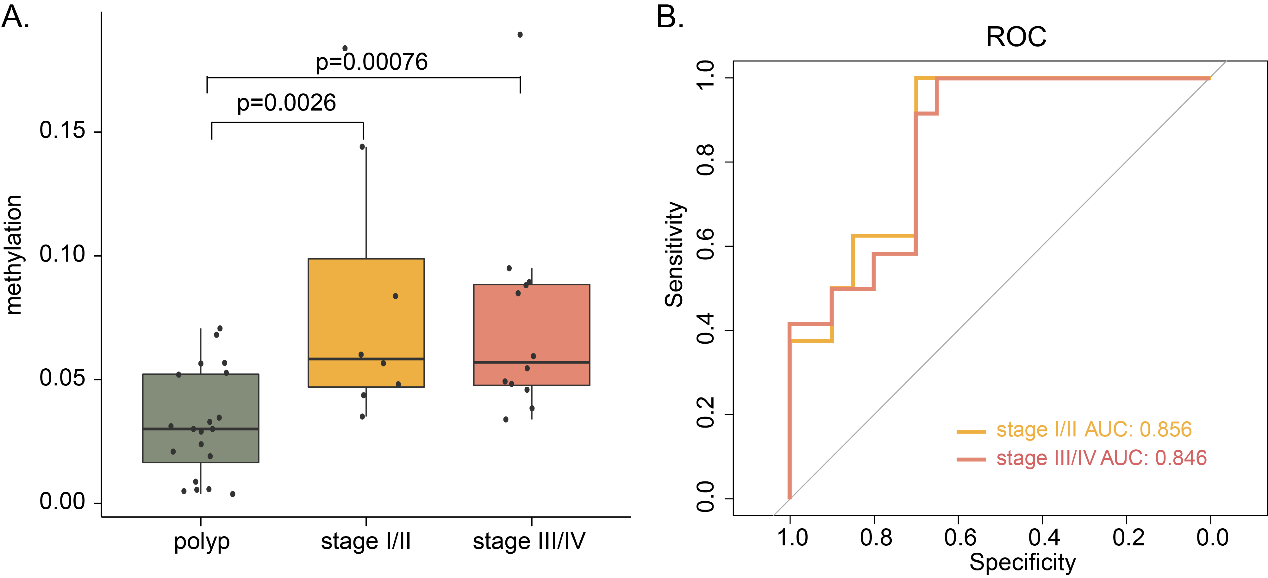


**Figure S7. Diagnostic power of cg27541454 cfDNA methylation in CRC with different clinical stages.** (A) The cfDNA methylation of cg27541454 in polyp and different stages of CRC. (B) ROC curves of cg27541454 cfDNA methylation for distinguishing CRC with stage I/II and stage (III/IV) from polyp, respectively.

**Supplementary Tables**

Supplementary Table S1. The 50 CpGs with strong discriminative power in the discovery tissue cohort

| **Probe** | **CHR** | **MAPINFO（hg19）** | **gene** | **DMC** | **AUC** |
| --- | --- | --- | --- | --- | --- |
| cg19300414 | 2 | 1746591 | PXDN | Hypo | 1 |
| cg22990430 | 8 | 56433632 | XKR4 | Hypo | 1 |
| cg27317433 | 19 | 1401781 | GAMT | Hyper | 1 |
| cg19389001 | 3 | 122640778 | SEMA5B | Hyper | 1 |
| cg02966153 | 19 | 1401767 | GAMT | Hyper | 1 |
| cg01878100 | 14 | 37666838 | MIPOL1 | Hypo | 1 |
| cg19966212 | 5 | 149792783 | CD74 | Hyper | 1 |
| cg16222762 | 18 | 40857464 | SYT4 | Hypo | 1 |
| cg02370667 | 16 | 84029511 | NECAB2 | Hyper | 1 |
| cg04651603 | 12 | 54321302 |  | Hypo | 1 |
| cg22546409 | 18 | 40857495 | SYT4 | Hypo | 1 |
| cg22663489 | 11 | 64107720 | CCDC88B | Hyper | 0.984375 |
| cg09638407 | 3 | 142839022 | CHST2 | Hyper | 0.96875 |
| cg07682037 | 11 | 63974153 | FERMT3 | Hyper | 0.96875 |
| cg18909638 | 14 | 37666810 | MIPOL1 | Hypo | 0.96875 |
| cg09924085 | 18 | 40857482 | SYT4 | Hypo | 0.96875 |
| cg13907146 | 3 | 50243565 | SLC38A3 | Hyper | 0.96875 |
| cg21597595 | 2 | 5506228 |  | Hypo | 0.96875 |
| cg09597070 | 12 | 54088972 |  | Hyper | 0.953125 |
| cg09826019 | 17 | 4802532 | C17orf107 | Hyper | 0.953125 |
| cg07834574 | 17 | 4802847 | CHRNE | Hyper | 0.953125 |
| cg26263766 | 19 | 58739734 | ZNF544 | Hypo | 0.953125 |
| cg13040921 | 19 | 33863415 | CEBPG | Hyper | 0.953125 |
| cg13079094 | 17 | 4802828 | CHRNE | Hyper | 0.953125 |
| cg17639623 | 22 | 50628803 | TRABD | Hyper | 0.953125 |
| cg05355311 | 22 | 50629224 | TRABD | Hyper | 0.953125 |
| cg17108819 | 2 | 87017953 | CD8A | Hyper | 0.9375 |
| cg01057920 | 13 | 111767872 | ARHGEF7-AS2 | Hyper | 0.9375 |
| cg06712559 | 1 | 968395 | AGRN | Hyper | 0.9375 |
| cg04260368 | 19 | 1401775 | GAMT | Hyper | 0.9375 |
| cg24080793 | 17 | 62097951 | ICAM2 | Hyper | 0.9375 |
| cg16047144 | 17 | 62097953 | ICAM2 | Hyper | 0.9375 |
| cg18987335 | 2 | 177039862 |  | Hyper | 0.9375 |
| cg26451062 | 19 | 33863417 | CEBPG | Hyper | 0.9375 |
| cg14482741 | 17 | 4802906 | CHRNE | Hyper | 0.9375 |
| cg27541454 | 1 | 975551 | AGRN | Hyper | 0.9375 |
| cg05236677 | 8 | 99952217 |  | Hyper | 0.9375 |
| cg03512945 | 14 | 101867429 |  | Hypo | 0.9375 |
| cg16294310 | 22 | 50629110 | TRABD | Hyper | 0.9375 |
| cg12097313 | 22 | 50628981 | TRABD | Hyper | 0.9375 |
| cg04495491 | 22 | 43067559 |  | Hyper | 0.921875 |
| cg20814095 | 17 | 4802981 | CHRNE | Hyper | 0.921875 |
| cg12506775 | 10 | 5734571 | C10orf18 | Hyper | 0.90625 |
| cg00557354 | 13 | 111767899 | ARHGEF7 | Hyper | 0.90625 |
| cg11235391 | 8 | 49343007 |  | Hypo | 0.90625 |
| cg14894823 | 12 | 58026592 | B4GALNT1 | Hypo | 0.90625 |
| cg18840956 | 5 | 95769005 | PCSK1 | Hypo | 0.90625 |
| cg19463976 | 6 | 27173633 |  | Hypo | 0.90625 |
| cg04486886 | 5 | 56784195 |  | Hypo | 0.90625 |
| cg13539460 | 19 | 46854076 | PPP5C | Hyper | 0.90625 |

Supplementary TableS2. The sequence information on 47 CpGs in the range of 25bp upstream and downstream.

Supplementary TableS3. The processed tissue MethylTarget sequencing data.

Supplementary TableS4. The processed cfDNA MethylTarget sequencing data.
